# Diabetes Management and Outcomes among Patients with Type 2 Diabetes Attending a Renal Service

**DOI:** 10.1155/2023/1969145

**Published:** 2023-04-26

**Authors:** Elizabeth Skalkos, Rohit Rajagopal, David Simmons

**Affiliations:** ^1^School of Medicine, Western Sydney University, Sydney, Australia; ^2^Macarthur Diabetes Service, Sydney, Australia

## Abstract

**Background:**

Patients with comorbid type 2 diabetes mellitus (T2DM) and renal disease, particularly those treated with insulin, often require complex pharmacological treatment and management of other diabetes complications.

**Aims:**

To assess the achievement of metabolic targets and compare the current management of renal service attenders with insulin- and noninsulin-treated T2DM.

**Methods:**

Single-centre retrospective cross-sectional study involving medical record review of patients with T2DM aged ≥18 years who visited a metropolitan renal outpatient clinic in 2017. Univariable analysis and multivariable logistic regression were used to identify factors associated with insulin treatment.

**Results:**

Among 268 patients (45.5% insulin-treated), mean HbA1c was higher in insulin-treated vs. noninsulin-treated patients (8.0 ± 1.8% (64 mmol/mol) vs. 6.8 ± 1.2% (51 mmol/mol), *p* < 0.001). Significantly fewer insulin-treated patients had HbA1c ≤ 7.0% (53 mmol/mol; 31.8% vs. 69.3%, *p* < 0.001). More insulin-treated patients had ischaemic heart disease (46.7% vs. 33.6%, *p* = 0.028), diabetic foot disease (15.6% vs. 4.8%, *p* = 0.003), retinopathy (40.2% vs. 11.0%, *p* < 0.001), and emergency attendance for severe hypoglycaemia (3.8% vs. 0% *p* = 0.042). Insulin treatment was more associated with chronic kidney disease stages 4-5 (adjusted odds ratio (aOR) 2.41, 95% CI 1.07-5.43), retinopathy (aOR 3.10, 95% CI 1.04-9.27), and podiatry review (aOR 5.06, 95% CI 1.20-21.38). Only 38 (14.2%) individuals were seen by a colocated public multidisciplinary diabetes service in 2017.

**Conclusions:**

Renal clinic attenders with T2DM, particularly if insulin-treated, remained at increased risk of diabetes-related complications, including severe hypoglycaemia, with limited input from the colocated hospital diabetes team. Approaches to increase coordination of diabetes care among renal patients should be investigated.

## 1. Introduction

A major complication of diabetes mellitus is end-stage renal disease (ESRD). Diabetes is the leading cause of ESRD in Australia, accounting for 38% of new cases in 2017 [1]. Furthermore, 50% of those with severe chronic kidney disease (CKD) from any cause requiring renal service attention have diabetes [1]. Morbidity from diabetes-related complications, including diabetic foot disease and retinopathy, is particularly high when CKD is present among people with type 2 diabetes mellitus (T2DM) [2, 3]. There is also an eightfold increased risk of cardiovascular and all-cause mortality, and as CKD progresses, the risk of acute events including severe hypoglycaemia increases [2]. Renal impairment in T2DM is independently associated with the reduced achievement of metabolic targets and complicates treatment choices due to interference with glucose metabolism, impaired renal clearance, and the metabolism of medications [4, 5]. Clearly, those with T2DM and renal disease have an increased complexity of care derived from greater morbidity and mortality, difficulty in achieving metabolic targets, and adjusting pharmacological treatments.

Standards for evidence-based care for patients with T2DM have been defined in the Royal Australian College of General Practitioners (RACGP) guidelines, the National Institute for Health and Care Excellence (NICE) guideline NG28 from the United Kingdom (UK), and the American Diabetes Association (ADA) Standards of Medical Care in Diabetes [6–8]. The Kidney Disease Improving Global Outcomes (KDIGO) Diabetes in CKD guidelines also have specific recommendations for individuals with comorbid diabetes and CKD [2]. The guideline recommendations for those patients with CKD include tight glycaemic control (HbA1c ≤ 7.0%), blood pressure (BP) (≤130/80 mmHg), and lipid control (total cholesterol < 4.0 mmol/L; LDL <2.0 mmol/L), risk factor reduction, and renin-angiotensin-aldosterone system (RAAS) blockade. Achievement of metabolic targets, as defined by RACGP guidelines, reduces the risk or slows the progression of CKD in T2DM [6, 9, 10].

Insulin therapy is an important therapeutic option for individuals with advanced CKD, although such treatment has shown mixed efficacy in achieving glycaemic control in T2DM [2, 11–13]. Insulin therapy is associated with weight gain and a greater risk of hypoglycaemia, both of which are associated with increased cardiovascular and other risks [2, 11, 12].

Diabetes mellitus is a significant problem in South Western Sydney (SWS) with a prevalence of 6.7%, compared with a national prevalence of 5.1% [14]. As a result, T2DM and its complications are a major strain on local hospital services. In view of the complexities in the care of those with insulin-treated T2DM, the aims of this study were to compare the local achievement of metabolic targets, diabetes management, and associated risks between renal patients with insulin- and noninsulin-treated T2DM.

## 2. Materials and Methods

This was a single-centre retrospective cross-sectional study among patients with diabetes mellitus attending an outpatient renal specialist service in SWS. The renal service is on the same hospital site as a major public multidisciplinary diabetes outpatient service. The diabetes service is staffed by endocrinologists, diabetes educators, podiatrists, and dieticians. Patients of the renal service have their diabetes mellitus managed by a mix of general practitioners (GPs) and private and public diabetes specialist care at the discretion of the GP and the person with diabetes.

The study was approved by the Quality Improvement Committee (reference: CT01_2019) of the South Western Sydney Local Health District Health Research Ethics Committee.

### 2.1. Data Collection

Deidentified quantitative data of all attending patients with diabetes mellitus aged ≥18 years between January 1st and December 31st, 2017 were collected following the review of electronic and paper medical records from both the local renal and diabetes services. Data collected included patient demographics, medications, clinical encounters, comorbidities, anthropometric measures, and metabolic parameters. Ethnicity was not ascertained.

The last result in 2017 by the date of collection was recorded for laboratory examinations. Where available, all estimated glomerular filtration rate (eGFR) and urinary albumin-to-creatinine ratio (UACR) data were recorded for the previous three years. The stage of CKD was determined based on the last recorded eGFR. Metabolic targets were defined in accordance with the RACGP guidelines (Supplementary Table 1), and body mass index (BMI) was defined by the World Health Organisation classification [6, 15]. Medications were recorded according to the last documented regimen. Attendance at a public diabetes service was recorded if it occurred in 2017 and included, but was not limited to the local hospital diabetes service. Attendance at public allied health services including podiatry, dietetics, and diabetes educators was available from electronic records. Private clinical encounters were recorded when available in the documentation.

### 2.2. Statistics

As this was a pilot quality improvement activity including all clinic attenders in the index year, no power calculations were undertaken. Analyses were undertaken using SPSS, version 25 (SPSS Inc., Chicago, IL, USA). Categorical variables were described using numbers and percentages, and Pearson's chi-squared test or Fischer's exact test, where appropriate, was used to compare groups. Continuous variables were described using mean ± standard deviation or median and range. Differences between groups were compared using the independent *t*-test. When skewed, the Mann–Whitney test or geometric mean was used. Stepwise binomial logistic regression including the variables with a significant difference in the univariable analysis was conducted to compare insulin- and noninsulin-treated patients. Nagelkerke's *R* square was computed for the proportion of variance accounted for in the model. Statistical significance at *p* < 0.05 was considered significant, and all statistical tests were two-tailed.

The rate of change in eGFR and UACR per year was calculated by subtracting the difference between recordings and dividing by the number of intervening years but did not take into account acute events leading to a temporary decline in eGFR. Those with ESRD in 2015 and those who received a transplant were excluded from calculating the rate of change in eGFR.

## 3. Results

**Figure 1:**
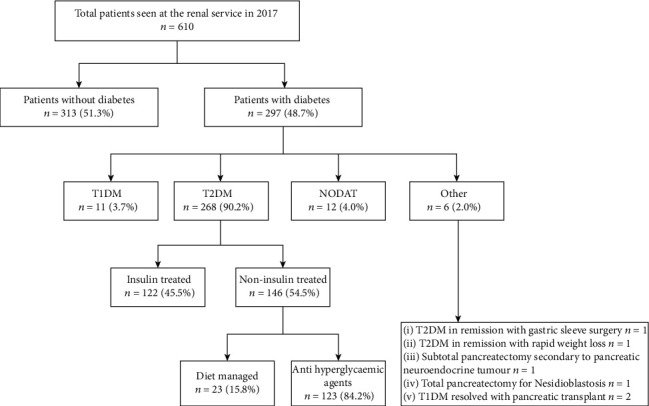
Classification of patients seen by the renal service. T1DM: type 1 diabetes mellitus; T2DM: type 2 diabetes mellitus; NODAT: new-onset diabetes after transplant.

Figure 1 shows the diabetes status of the 610 individuals seen by the renal service in 2017. Of the 297 (48.7%) with diabetes, 11 (3.7%) had type 1 diabetes mellitus, 268 (90.2%) had T2DM (122 (45.5%) insulin-treated), 12 (4.0%) had new-onset diabetes after transplant (NODAT), and 6 (2.0%) were classified as other.

**Figure 2:**
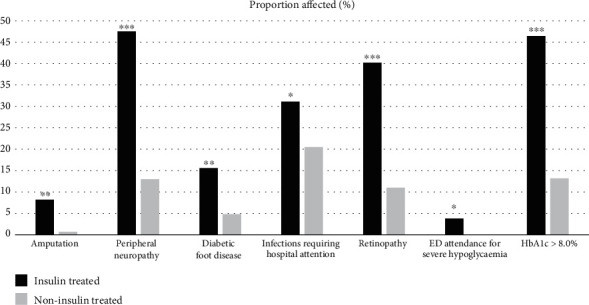
Key diabetes management issues—insulin- vs. noninsulin-treated patients. Insulin treated (black) vs. noninsulin treated (grey) ^∗^*p* < 0.05; ^∗∗^*p* < 0.01; ^∗∗∗^*p* < 0.001.

Table 1 shows the characteristics of all patients with T2DM, with a mean age of 69 ± 11 years, 63.4% were male and 44.9% had CKD stages 4-5. Most had hypertension (92.9%) and/or dyslipidaemia (75.0%). Within the cohort, 11.6% had renal biopsies. Table 1 also compares the clinical characteristics according to insulin treatment status. Insulin-treated individuals were younger (67 ± 10 vs. 70 ± 12 years, *p* = 0.019), had a longer duration of diabetes (17 vs. 8 years, *p* < 0.001), and more individuals with CKD stages 4-5 (55.7% vs. 35.7%, *p* = 0.001).  Figure 2 demonstrates that more insulin-treated patients had ischemic heart disease (46.7% vs. 33.6%, *p* = 0.028), dyslipidaemia (81.1% vs. 69.9%, *p* = 0.034), heart failure (18.9% vs. 10.3%, *p* = 0.045), history of amputation (8.2% vs. 0.7%, *p* = 0.002), peripheral neuropathy (47.5% vs. 13.0%, *p* < 0.001), diabetic foot disease (15.6% vs. 4.8%, *p* = 0.003), infections requiring hospital attention (31.1% vs. 20.5%, *p* = 0.047), retinopathy (40.2% vs. 11.0%, *p* < 0.001), and received dialysis (20.5% vs. 10.3%, *p* = 0.027). Those treated with insulin were more likely to experience severe hypoglycaemic events requiring emergency department (ED) attendance (3.8% vs. 0%, *p* = 0.042). Total ED visits, hospitalisations, and number of nephrology appointments were significantly higher in the insulin-treated group. Rates of hypertension, obstructive sleep apnoea, cerebrovascular accidents, peripheral vascular disease, cataracts, and depression were similar between the two groups.

Table 2 compares the metabolic target achievement according to insulin treatment status. The mean BMI was significantly higher in the insulin-treated group (35.0 ± 6.9 kg/m^2^ vs. 32.7 ± 7.3 kg/m^2^, *p* = 0.028). The most common metabolic target achieved overall was BP (57.0%), and BMI was the metabolic target achieved the least (9.4%). HbA1c ≤ 7.0% (53 mmol/mol) was achieved in 50.9%. Higher HbA1c was found in insulin-treated patients (mean HbA1c 8.0 ± 1.8% (64 mmol/mol) vs. 6.8 ± 1.2% (51 mmol/mol), *p* < 0.001) and significantly fewer insulin-treated patients met the metabolic target of HbA1c ≤ 7.0% (53 mmol/mol: 31.8% vs. 69.3%, *p* < 0.001). Mean systolic BP and diastolic BP, total cholesterol, triglycerides, and low-density lipoprotein were comparable between groups.

The rate of decline of eGFR per year was faster in the insulin-treated group (-5 mL/min/1.73 m^2^/year vs. -2 mL/min/1.73 m^2^/year, *p* < 0.001). The median UACR was higher in the insulin-treated group (76.3 (0.7; 812.7) mg/mmol vs. 14.0 (0.3; 1080) mg/mmol, *p* < 0.001), although there was no significant difference in the rate of change in UACR per year (-2.1 (-295.6; 273.0) vs. -0.2 (-580.7; 204), *p* = 0.611).

Table 3 shows that metformin and sulphonylureas were used significantly less among insulin-treated patients (30.3% vs. 45.9%, *p* = 0.009; 15.6% vs. 42.5%, *p* < 0.001, respectively). There were no significant differences in the use of antihypertensive agents based on insulin treatment status. Renin-angiotensin-aldosterone system (RAAS) blockers were prescribed to 70.1% of the cohort. There was a high use of statins, particularly among those with insulin-treated T2DM (86.1% vs. 69.9%, *p* = 0.002).

Dietetics were the most common allied health service encountered (Table 1). Overall, 38 (14.2%) individuals were seen by a public multidisciplinary diabetes service in 2017. Insulin-treated individuals were more likely to be engaged with a public diabetes service and attend public podiatry services (22.1% vs. 7.5%, *p* = 0.001; 17.2 vs. 3.4%, *p* < 0.001, respectively). Being engaged in a public diabetes service was associated with increased documented attendance with diabetes educators (21.1% vs. 2.2%, *p* < 0.001), dieticians (34.2% vs. 8.3%, *p* < 0.001), and podiatrists (34.2% vs. 5.2%, *p* < 0.001) (Supplemental Table 2). Similarly, these individuals were also more likely to have documented that they had an eye review by an ophthalmologist or optometrist in 2017 (28.9% vs. 7.4%, *p* < 0.001). Public diabetes service attenders were more likely to use insulin treatment (71.1% vs. 41.3%, *p* = 0.001), dipeptidyl peptidase-4 inhibitors (DPP4i) (44.7% vs. 21.3%, *p* = 0.002), and glucagon-like peptide-1 receptor agonists (GLP-1 RA) (13.2% vs. 2.6%, *p* = 0.002).

The logistic regression model explained 45.5% of the variance comparing insulin- and noninsulin-treated patients.  Table 4 shows that compared with noninsulin-treated patients and after adjustment, those receiving insulin therapy were 2-3-fold more likely to have CKD stages 4-5, retinopathy, peripheral neuropathy, and 5.06-fold (95% CI 1.20-21.38) more to have undergone podiatry review. There was a 73% (95% CI 29-131) increase in insulin treatment for every 1% (11 mmol/mol) increase in HbA1c.

## 4. Discussion

We have shown in this first Australian study, and one of few international studies, that compared with noninsulin-treated renal patients with T2DM, those treated with insulin had worse hyperglycaemia, more hypoglycaemia, and experienced a greater burden of diabetes-related complications including foot problems, hospitalisation, and a faster decline in renal function. There were low rates of documented diabetes risk monitoring, and few patients were documented to be receiving care from the local multidisciplinary diabetes service, while those that did, received more modern pharmacotherapy. The study was undertaken to understand whether renal services might benefit from working more closely with colocated diabetes specialist services. Our findings suggest that such coworking is likely to be beneficial.

Previous studies comparing glucose-related outcomes between insulin- and noninsulin-treated patients with diabetes mellitus and CKD found a mean HbA1c of 7.5% (58 mmol/mol) among insulin-treated patients compared to 6.7% (50 mmol/mol) among those who were noninsulin-treated [13]. Without stratifying by insulin treatment status, other cohort studies among patients with CKD and T2DM have demonstrated a mean HbA1c of 6.7-8.6% (50-70 mmol/mol), with the proportion achieving HbA1c ≤ 7.0% (53 mmol/mol) ranging from 23.9 to 45.8% and HbA1c ≤ 8.0% (64 mmol/mol) from 68.1 to 75.0% [3, 16–24].

Insulin treatment is a major risk factor for hypoglycaemia in those with CKD, and less stringent HbA1c targets may be appropriate in individuals who experience hypoglycaemia in view of its association with increased mortality [6, 25]. Additionally, higher individualised HbA1c targets may be appropriate in individuals who are older, with a longer duration of diabetes and severe established vascular complications, as is reflected in the insulin-treated cohort [5]. Clinical inertia including delay or failure to intensify insulin treatment may also play a role in the suboptimal glycaemic control found in this study [26].

That diabetes care can be improved is suggested by those attending the public diabetes service being more likely to be treated with modern diabetes treatment including DPP4i and GLP-1 RA, which are beneficial in reducing the risk of hypoglycaemia. Nevertheless, there was a relatively low uptake of sodium-glucose cotransporter 2 inhibitors (SGLT2i) which are now considered first-line therapy in combination with metformin for individuals with eGFR ≥ 30 mL/min/1.73 m^2^ due to their reduced risk of renal and cardiovascular morbidity and mortality [2, 8, 10, 27]. These recommendations, however, date more recently than this study, and thus we would expect a future comparative study to reflect the changes in antihyperglycaemic therapy recommendations.

In contrast to the relatively low achievement of glycaemic targets, our cohort of patients was more likely to achieve the BP target than those in earlier studies, albeit with room for improvement. Hypertension is a significant contributor to the development of renal disease and its progression [19, 23]. Sustained blood pressure control is the single most effective intervention to reduce the risk and progression of CKD and simultaneously reduce cardiovascular risk [28]. Individuals with comorbid T2DM and CKD usually achieve worse blood pressure control than those without CKD, notwithstanding the greater use of antihypertensive agents [4]. Cohort studies of patients with T2DM and CKD have shown that 14.3-20.8% of patients achieved BP ≤ 130/80 mmHg, 20.8-41.0% achieved ≤140/80 mmHg, and 38.8-60.7% achieved BP ≤ 140/90 mmHg [17, 18, 20, 21, 23]. There were insufficient data to identify the reasons behind this better BP control in our study. Studies of the epidemiology of hypertension in the background population and any wider secular trends such as mortality could be helpful. This discordance between the achievement of blood pressure and glycaemic targets in T2DM has recently been shown elsewhere, with the suggestion that glycaemic (vs. blood pressure) management may warrant greater inclusion of diabetes specialist service intervention [29].

The RACGP, KDIGO, and ADA recommend patients with T2DM and CKD use RAAS blockers as they have been shown to slow the progression of renal disease [2, 6, 8]. The uptake of RAAS blockade (70.1%) in this study was similar to that in other cohorts of patients with CKD (49.0-79.7%), except for one French cohort with T2DM and CKD which showed uptake as high as 91.5% [17–20, 22, 23, 30]. Higher uptake in this French cohort may be related to the exclusion of patients with stage 5 CKD [23]. Participation in integrated multidisciplinary care has shown improvements in RAAS blocker uptake [18, 31].

Renal specialist services can significantly slow the progression of CKD, improve survival, and control blood pressure while increasing the uptake of RAAS blockers and statins among patients with T2DM [32, 33]. However, achievement of metabolic targets (set to reduce morbidity) and management of other complications including acute foot events, retinopathy, and hypoglycaemia would likely have benefitted from coordination with diabetes specialist services. Further, there were low rates of documented diabetes risk monitoring, although information regarding private health encounters was not available beyond the correspondence that had been received by the renal physician. Renal physicians may either not have access to or not have documented this information. Lack of access and documentation are both indicators of fragmented care, potentially predisposing to duplication of tests and treatments, conflicting messages to patients, multiple changes to treatment plans, and may even result in conditions being undiagnosed and/or untreated [34]. This raises the question of whether there is a need for better coordination between renal and diabetes services.

One way to reduce risks and enhance coordination between renal and diabetes care may be to establish a joint renal-diabetes clinic, involving multidisciplinary endocrinology and nephrology specialist care, which have demonstrated improvements in achieving metabolic targets and slowing the progression of renal disease [18, 20, 31]. A qualitative evaluation of one such service in Australia has shown improved integration of care and perception of improved health and management of health [35]. Evaluation of metabolic and complication outcomes is awaited. Developing a similar model of care locally, particularly targeting those receiving insulin treatment, is likely to improve the way diabetes care is being delivered. Other models of care to consider include self-management programmes for those with comorbid diabetes and renal disease. These have demonstrated improved glycaemic control, maintenance of renal function, and improvements in quality of life [36]. Case conferencing with specialists and upskilling GPs may also play a role, considering that large proportions of those with comorbid T2DM and CKD are seen in primary care [37].

The strengths of the study are being the first in Australia to explore the achievement of metabolic targets and assess the management of patients with T2DM and CKD based on insulin treatment status. Further strengths are the inclusion of a complete clinic cohort with a modest catchment population size, in alignment with other similar studies [21, 22, 24, 38]. Additionally, the data collected includes a comprehensive register of measurable diabetes care-related parameters and reflects real-world clinical practice.

Limitations of this study include that it is a single-centre study, relaying data specific to an Australian healthcare system which may restrict its relevance to other centres. Due to the retrospective cross-sectional study design, there were missing data, and causality was not able to be determined. Private health encounters may not have all been recorded, underestimating the number of individuals engaged in diabetes risk monitoring. As a result of the comanagement of diabetes, it is difficult to assess who and what was contributed by the varying healthcare professionals. Additionally, the data were not adjusted for time spent under the renal service, and this may affect the management of their diabetes and CKD. The potential inaccuracy of HbA1c measurement in CKD should also be noted. This occurs secondary to rapid cell turnover and may result in falsely lower HbA1c values [39]. Nevertheless, HbA1c remains preferable to other measurements including fructosamine and glycated albumin [39].

It would be useful to conduct another study in this cohort in the future to determine the effects of changing clinical practices, including the routine use of SGLT2 inhibitors as first-line therapy. It may be useful to assess the influence of individual nephrologists on the diabetes care and management of patients by comparing their initial nephrologist consultation to a point in time in the future. Additionally, the efficacy of joint diabetes-renal clinics in an Australian healthcare setting needs to be evaluated.

## 5. Conclusion

Renal clinic attendees with insulin-treated T2DM had a higher burden of diabetes-related complications including diabetic foot disease, retinopathy, hypoglycaemia, and hospitalisation. In contrast to good blood pressure control, glycaemic management, monitoring of diabetes risk, and use of more modern pharmacotherapy were limited. A joint renal diabetes clinic may be one way to reduce T2DM-related morbidity and mortality. Studies are needed to assess the efficacy of such joint clinics in the Australian healthcare system.

## Figures and Tables

**Table 1:** Clinical characteristics of patients with type 2 diabetes both overall and according to insulin treatment status.

Characteristic	Total (*n* = 268)	Insulin treated (*n* = 122)	Noninsulin treated (*n* = 146)	*p*
Age (years), mean ± SD	69 ± 11	67 ± 10	70 ± 12	0.019
Male, *n* (%)	170 (63.4)	79 (64.8)	91 (62.3)	0.681
Aboriginal and/or Torres Strait Islander, *n* (%)	7 (2.6)	2 (1.6)	5 (3.4)	0.361
Current smoker^a^, *n* (%)	26 (10.4)	12 (10.5)	14 (10.3)	0.952
Known duration of T2DM (years)^b^, geometric mean	12	17	8	<0.001^∗^
Chronic kidney disease, *n* (%)	265 (98.9)	122 (100.0)	143 (97.9)	0.003^∗^
Stage 1	13 (4.9)	2 (1.6)	11 (7.5)	
Stage 2	29 (10.8)	7 (5.7)	22 (15.1)	
Stage 3a	40 (14.9)	18 (14.8)	22 (15.1)	
Stage 3b	64 (23.9)	27 (22.1)	37 (25.3)	
Stage 4	67 (25.0)	35 (28.7)	32 (21.9)	
Stage 5/ESRD	52 (19.4)	33 (27.0)	19 (13.0)	
Missing	3 (1.1)	0 (0)	3 (2.1)	
Stages 1-3	146 (55.1)	54 (44.3)	92 (64.3)	0.001^∗^
Stages 4-5	119 (44.9)	68 (55.7)	51 (35.7)	
Renal biopsy, n (%)	31 (11.6)	18 (14.8)	13 (8.9)	0.136
Comorbidities				
Hypertension	249 (92.9)	116 (95.1)	133 (91.1)	0.205
Ischaemic heart disease	106 (39.6)	57 (46.7)	49 (33.6)	0.028^∗^
Dyslipidaemia	201 (75.0)	99 (81.1)	102 (69.9)	0.034^∗^
Obstructive sleep apnoea	59 (22.0)	33 (27.0)	26 (17.8)	0.069
Cerebrovascular accident	36 (13.4)	17 (13.9)	19 (13.0)	0.826
Heart failure	38 (14.2)	23 (18.9)	15 (10.3)	0.045^∗^
Currently on dialysis	40 (14.9)	25 (20.5)	15 (10.3)	0.027^∗^
Kidney transplantation	5 (1.9)	4 (3.3)	1 (0.7)	0.181†
Peripheral vascular disease	52 (19.4)	26 (21.3)	26 (17.8)	0.470
Amputation	11 (4.1)	10 (8.2)	1 (0.7)	0.002^∗^
Peripheral neuropathy	77 (28.7)	58 (47.5)	19 (13.0)	<0.001^∗^
Autonomic neuropathy	4 (1.5)	3 (2.5)	1 (0.7)	0.333†
Diabetic foot disease	26 (9.7)	19 (15.6)	7 (4.8)	0.003^∗^
Infections requiring hospital attention	68 (25.4)	38 (31.1)	30 (20.5)	0.047^∗^
Retinopathy	65 (24.3)	49 (40.2)	16 (11.0)	<0.001^∗^
Cataracts	61 (22.8)	28 (23.0)	33(22.6)	0.946
Depression	39 (14.6)	19 (15.6)	20 (13.7)	0.665
ED attendance for severe hypoglycaemia	4 (1.5)	4 (3.8)	0 (0)	0.042^∗^†
Service				
Number of nephrology appointments attended				
Mean ± SD	2.4 ± 1.7	2.8 ± 2.1	2.1 ± 1.1	0.001^∗^‡
Median (range)	2 (1-16)	2 (1-16)	2 (1-6)	
Public diabetes service	38 (14.2)	27 (22.1)	11 (7.5)	0.001^∗^
Diabetes educator	13 (4.9)	9 (7.4)	4 (2.7)	0.078
Dietician	32 (11.9)	18 (14.8)	14 (9.6)	0.194
Podiatry	26 (9.7)	21 (17.2)	5 (3.4)	<0.001 ^∗^
Eye review	28 (10.4)	16 (13.1)	12 (8.2)	0.192
Number of emergency department visits				
Mean ± SD	1.1 ± 1.7	1.4 ± 1.9	0.9 ± 1.5	0.007^∗^‡
Median (range)	0 (0-11)	1 (0-11)	0 (0-8)	
Number of hospital admissions				
Mean ± SD	0.9 ± 1.5	1.0 ± 1.4	0.8 ± 1.6	0.029^∗^‡
Median (range)	0 (0-10)	0 (0-5)	0 (0-10)	

Pearson's chi-square test was used for categorical variables, and independent *t*-test was used for continuous variables. †Fisher's exact test. ‡Mann–Whitney test. ^a^*n* = 18 (6.7%) of data unavailable. ^b^*n* = 64 (23.9%) of data unavailable. ED: emergency department.

**Table 2:** Metabolic target and renal parameters data according to insulin treatment status.

	Total	Insulin treated	Noninsulin treated	*p*
HbA1c				
Mean ± SD (%; mmol/mol)	7.4 ± 1.6; 57	8.0 ± 1.8; 64	6.8 ± 1.2; 51	<0.001^∗^
By CKD stage, mean ± SD (%; mmol/mol)				0.770^§^
Stage 1	7.0 ± 1.3; 53			
Stage 2	7.7 ± 1.3; 61			
Stage 3a	7.6 ± 1.9; 60			
Stage 3b	7.4 ± 1.4; 57			
Stage 4	7.3 ± 1.6; 56			
Stage 5	7.5 ± 1.9; 58			
≤7.0% (53 mmol/mol), *n* (%)	114/224 (50.9)	35/110 (31.8)	79/114 (69.3)	<0.001^∗^
≤8.0% (64 mmol/Mol), *n* (%)	158/224 (70.5)	59/110 (53.6)	99/114 (86.8)	<0.001^∗^
SBP (mmHg), mean ± SD	131 ± 15	130 ± 15	131 ± 15	0.687
DBP (mmHg), mean ± SD	71 ± 7	71 ± 7	71 ± 7	0.738
BP ≤130/80 mmHg, *n* (%)	151/265 (57.0)	76/122 (62.3)	75/143 (52.4)	0.107
≤140/80 mmHg, *n* (%)	211/265 (79.6)	99/122 (81.1)	112/143 (78.3)	0.569
TC (mmol/L), mean ± SD	4.0 ± 1.1	4.0 ± 1.1	4.0 ± 1.1	0.571
<4.0 mmol/L, *n* (%)	111/199 (55.8)	52/96 (54.2)	59/103 (57.3)	0.658
TG (mmol/L), geometric mean	1.8	2.0	1.7	0.069
<2.0 mmol/L, *n* (%)	111/195 (56.9)	49/94 (52.1)	62/101 (61.4)	0.192
LDL (mmol/L), mean ± SD	2.0 ± 1.0	1.9 ± 0.8	2.2 ± 1.1	0.064
<2.0 mmol/L, *n* (%)	69/130 (53.1)	35/64 (54.7)	34/66 (51.5)	0.717
HDL (mmol/L), mean ± SD	1.07 ± 0.36	0.98 ± 0.26	1.16 ± 0.42	0.001^∗^
≥1.0 mmol/L, *n* (%)	89/159 (56.0)	37/83 (44.6)	52/76 (68.4)	0.002^∗^
BMI (kg/m^2^), mean ± SD	33.8 ± 7.2	35.0 ± 6.9	32.7 ± 7.3	0.028^∗^
18.5-24.9 kg/m^2^, *n* (%)	18/192 (9.4)	6/94 (6.4)	12/98 (12.2)	0.164
Renal parameters data				
eGFR (mL/min/1.73 m^2^), median (min; max)	33 (3; 90)	26 (3; 90)	38 (4; 90)	<0.001^∗^
Rate of change eGFR/year (mL/min/1.73 m^2^/year), median (min; max)	-3.0 (-64; 28)	-5.0 (-64; 22)	-2.0 (-40; 28)	0.001‡^∗^
UACR (mg/mmol)				
Median (min; max)	23.4 (0.3; -1080)	76.3 (0.7; 812.7)	14.0 (0.3; 1080)	<0.001‡^∗^
Within normal range (women)				
<3.5 mg/mmol; men <2.5 mg/mmol)	30/159 (18.9)	5/72 (7.0)	25/87 (28.7)	<0.001^∗^
Rate of change UACR/year (mg/mmol/year), median (min; max)	-0.5 (-580.7; 273.0)	-2.1 (-295.6; 273.0)	-0.2 (-580.7; 204)	0.611‡
Haemoglobin (g/L), mean ± SD	122 ± 19	121 ± 19	124 ± 19	0.288

Independent *t*-test, unless otherwise stated. ^§^ANOVA. ‡Mann–Whitney test. HbA1c: glycated haemoglobin; SBP: systolic blood pressure; DBP: diastolic blood pressure; BP: blood pressure; TC: total cholesterol; TG: triglycerides; LDL: low density lipoprotein; HDL: high density lipoprotein; BMI: body mass index; eGFR: estimated glomerular filtration rate; UACR: urine albumin creatinine ratio; Hb: haemoglobin; min: minimum value; max: maximum value.

**Table 3:** Medications of patients according to insulin treatment status.

	Medications	Number of patients (%)			*p*
		Total	Insulin treated (*n* = 122)	Noninsulin treated (*n* = 146)	
Antihyperglycaemic agents	Metformin	104 (38.8)	37 (30.3)	67 (45.9)	0.009^∗^
	Sulphonylurea	81 (30.2)	19 (15.6)	62 (42.5)	<0.001^∗^
	DPP4i	66 (24.6)	24 (19.7)	42 (28.8)	0.085
	GLP-1 RA	11 (4.1)	5 (4.1)	6 (4.1)	0.996
	SGLT2i	15 (5.6)	7 (5.7)	8 (5.5)	0.927

Antihypertensive	RAAS blocker¶	188 (70.1)	89 (73.0)	99 (67.8)	0.360
	ACEi	61 (22.8)	31 (25.4)	30 (20.5)	0.344
	ARB	131 (48.9)	58 (47.5)	73 (50.0)	0.688
	CCB	133 (49.6)	59 (48.4)	74 (50.7)	0.705
	Beta blocker	122 (45.5)	59 (48.4)	63 (43.2)	0.394

Lipid lowering agents	Statin	207 (77.2)	105 (86.1)	102 (69.9)	0.002^∗^
	Fibrate	31 (11.6)	19 (15.6)	12 (8.2)	0.061
	Ezetimibe	38 (14.2)	18 (14.8)	20 (13.7)	0.805

Aspirin	Aspirin	96 (35.8)	50 (41.0)	46 (31.5)	0.107

Renal drugs	ESA	56 (20.9)	31 (25.4)	25 (17.1)	0.097

Pearson's chi-square test, unless otherwise stated. ^¶^Includes individuals receiving an ACEi or ARB. DPP4i: dipeptidyl peptidase-4 inhibitor; GLP-1 RA: glucagon-like peptide 1 agonist; SGLT2i: sodium glucose co-transporter 2 inhibitor; RAAS blocker: renin-angiotensin-aldosterone system blocker; ACEi: angiotensin converting enzyme inhibitor; ARB: angiotensin receptor blocker; CCB: calcium channel blocker; ESA: erythropoietin stimulating agent.

**Table 4:** Binary logistic regression analysis comparing adjusted odds ratios for factors associated with insulin treatment.

Variables	Adjusted odds ratio (95% confidence interval)	*p*
CKD stages 4-5	2.41 (1.07-5.43)	0.035^∗^
Per 1% (11 mmol/mol) rise in HbA1c	1.73 (1.29-2.31)	<0.001^∗^
Retinopathy	3.10 (1.04-9.27)	0.043^∗^
Peripheral neuropathy	3.09 (1.14-8.42)	0.027^∗^
Podiatry review in 2017	5.06 (1.20-21.38)	0.028^∗^

Variables entered included age, sex, current smoker, duration of diabetes (<10 years or ≥10 years), last HbA1c (%/per 11 mmol/mol), presence of retinopathy, peripheral neuropathy, being on a statin, and having a podiatry review in 2017.

## Data Availability

Access to data is restricted. The data and distribution of this audit have not been approved under the terms of the ethics approval.

## Supplementary Materials

Supplementary Materials Table 1: the Royal Australian College of General Practitioners (RACGP) clinical management goals (2022) and World Health Organisation (WHO) body mass index (BMI) target (2021). Table 2: attendance at public diabetes clinic vs. variables.
