# Supplementary material for: Diabetes Management and Outcomes among Patients with Type 2 Diabetes Attending a Renal Service
Source: J Diabetes Res. 2023 Apr 26;2023:1969145. doi: 10.1155/2023/1969145 (PMC10156453; doi:10.1155/2023/1969145)
Supplement: Supplementary Materials — Table 1: the Royal Australian College of General Practitioners (RACGP) clinical management goals (2022) and World Health Organisation (WHO) body mass index (BMI) target (2021). Table 2: attendance at public diabetes clinic vs. variables. [file 1969145.f1.docx]

**Supplemental table 1: The Royal Australian College of General Practitioners (RACGP) clinical management goals (2022) and World Health Organisation (WHO) Body mass index (BMI) target (2021) (6, 15)**

| **Metabolic parameter** | **Target** |
| --- | --- |
| HbA1c | ≤7% (53 mmol/mol) |
| Total cholesterol | <4.0mmol/L |
| Triglycerides | <2.0mmol/L |
| Low density lipoprotein (LDL) | <2.0mmol/L |
| High density lipoprotein (HDL) | ≥1.0mmol/L |
| Blood pressure in those with diabetes and albuminuria | ≤130/80mmHg |
| Urine albumin-to-creatinine ratio (UACR) | Women: <3.5mg/mmol  Men: <2.5mg/mmol |
| Body mass index (BMI) | 18.5-24.9kg/m^2^ |

**Supplemental table 2**

| *Attendance at public diabetes clinic vs variables* | | | | |
| --- | --- | --- | --- | --- |
|  | **Variable** | **Attended public diabetes service (n=38)** | **Did not attend public diabetes service (n=230)** | ***p*** |
| Demographics | **Male gender,** n (%) | 30 (78.9) | 140 (60.9) | **0.032** |
|  | **Age (years),** mean ± SD | 70 ± 10 | 68 ± 11 | 0.458 |
|  | **Known duration of T2DM^a^ (years),** geometric mean | 12 | 12 | 0.880 |
|  | **Current smoker^b^,** n (%) | 2 (5.9) | 24 (11.1) | 0.353 |
|  | **Aboriginal or Torres Strait Islander,** n (%) | 1 (2.6) | 6 (2.6) | 0.993 |
|  | **Chronic kidney disease (CKD),** n (%)  Stage 1-3  Stage 4-5 | 20 (52.6)  18 (47.4) | 126 (54.8)  101 (43.9) | 0.742 |
|  | **Renal biopsy,** n (%) | 7 (18.4) | 31 (13.5) | 0.154 |
| Comorbidities, n (%) | **Hypertension** | 34 (89.5) | 215 (93.5) | 0.373 |
|  | **IHD** | 15 (39.5) | 91 (39.6) | 0.991 |
|  | **Dyslipidaemia** | 34 (89.5) | 167 (72.6) | **0.026** |
|  | **Obstructive sleep apnoea** | 8 (21.1) | 51 (22.2) | 0.877 |
|  | **Cerebrovascular accident** | 5 (13.2) | 31 (13.5) | 0.957 |
|  | **Heart failure** | 5 (13.2) | 33 (14.3) | 0.846 |
|  | **Currently on dialysis** | 3 (7.9) | 37 (16.1) | 0.189 |
|  | **Peripheral vascular disease** | 10 (26.3) | 42 (18.3) | 0.245 |
|  | **Amputation** | 2 (5.3) | 9 (3.9) | 0.698 |
|  | **Peripheral neuropathy** | 11 (28.9) | 66 (28.7) | 0.975 |
|  | **Autonomic neuropathy** | 0 (0) | 4 (1.7) | 0.413† |
|  | **Diabetic foot disease** | 4 (10.5) | 22 (9.6) | 0.853 |
|  | **Infections requiring hospital attention** | 8 (21.1) | 60 (26.1) | 0.509 |
|  | **Retinopathy** | 10 (26.3) | 55 (23.9) | 0.749 |
|  | **ED attendance for severe hypoglycaemia** | 0 (0) | 4 (1.7) | 0.413† |
|  | **Depression** | 4 (10.5) | 35 (15.2) | 0.447 |
| Metabolic targets and renal markers | **HbA1c**  Mean ± SD (%)  Mean (mmol/mol)  ≤7.0%, n (%)  ≤8.0%, n (%) | 7.7 ± 1.7  61  16/37 (43.2)  136/187 (72.7) | 7.4 ± 1.6  57  98/187 (52.4)  22/37 (59.5) | 0.185  0.308  0.106 |
|  | **SBP,** mean ± SD  **DBP,** mean ± SD  **BP** ≤130/80mmHg, n (%)  ≤140/80mmHg, n (%) | 131 ± 16  71 ± 7  21/38 (55.3)  28/38 (73.7) | 131 ± 15  71 ± 7  130/227 (57.3)  183/227 (80.6) | 0.984  0.949  0.817  0.326 |
|  | **TC,** mean ± SD  < 4.0 mmol/L, n (%) | 4.0 ± 1.0  16/31 (51.6) | 4.0 ± 1.1  95/168 (56.5) | 0.438  0.611 |
|  | **TG,** geometric mean  < 2.0 mmol/L, n (%) | 2.3  16/31 (51.6) | 2.1  95/164 (57.9) | 0.438  0.515 |
|  | **LDL**, mean ± SD  < 2.0 mmol/L, n (%) | 2.1 ± 0.8  9/24 (37.5) | 2.0 ± 1.0  60/106 (56.6) | 0.863  0.090 |
|  | **HDL,** mean ± SD  ≥ 1 mmol/L, n (%) | 0.88 ± 0.17  9/28 (32.1) | 1.11 ± 0.37  80/131 (61.1) | **<0.001**  **0.005** |
|  | **BMI,** mean ± SD  18.0-24.9kg/m^2^ | 34.2 ± 6.4  2/28 (7.1) | 33.7± 7.3  16/164 (9.8) | 0.747  0.661 |
|  | **UACR (mg/mmol)**  Median (min; max)  Within normal range (women <3.5mg/mmol; men <2.5mg/mmol) | 62.6 (0.6; 470)  4/28 (14.3) | 20.4 (0.3; 1080)  26/131 (19.8) | 0.989  0.495 |
| Clinical encounters in 2017 | **Number of nephrology appointments attended**  Mean ± SD  Median (range) | 2.6 ± 1.3  2 (1- 7) | 2.4 ± 1.7  2 (1- 16) | 0.409  0.091^‡^ |
|  | **Number of emergency department visits**  Mean ± SD  Median (range) | 0.8 ± 1.3  0 (0- 5) | 1.2 ± 1.8  0 (0- 11) | 0.250^‡^ |
|  | **Number of hospital admissions**  Mean ± SD  Median (range) | 0.8 ± 1.4  0 (0- 7) | 0.9 ± 0.9  0 (0- 10) | 0.518^‡^ |
|  | **Diabetes educator**, n (%) | 8 (21.1) | 5 (2.2) | **<0.001** |
|  | **Dietician,** n (%) | 13 (34.2) | 19 (8.3) | **<0.001** |
|  | **Podiatry**, n (%) | 13 (34.2) | 12 (5.2) | **<0.001** |
|  | **Eye review**, n (%) | 11 (28.9) | 17 (7.4) | **<0.001** |
| Medications, n (%) | **Insulin** | 27 (71.1) | 95 (41.3) | **0.001** |
|  | **Metformin** | 15 (39.5) | 89 (38.7) | 0.927 |
|  | **Sulphonylurea** | 13 (34.2) | 68 (29.6) | 0.563 |
|  | **DPP4i** | 17 (44.7) | 49 (21.3) | **0.002** |
|  | **GLP-1 RA** | 5 (13.2) | 6 (2.6) | **0.002** |
|  | **SGLT2i** | 2 (5.3) | 13 (5.7) | 0.923 |
|  | **ACEi** | 9 (23.7) | 52 (22.6) | 0.884 |
|  | **ARB** | 19 (50.0) | 112 (48.7) | 0.882 |
|  | **RAAS blocker^¶^** | 28 (73.7) | 160 (69.6) | 0.471 |
|  | **CCB** | 18 (47.4) | 115 (50.0) | 0.764 |
|  | **Frusemide** | 12 (31.6) | 66 (28.7) | 0.717 |
|  | **Beta blocker** | 16 (42.1) | 106 (46.1) | 0.648 |
|  | **Statin** | 34 (89.5) | 173 (75.2) | 0.052 |
|  | **Fibrate** | 7 (18.4) | 24 (10.4) | 0.154 |
|  | **Ezetimibe** | 6 (15.8) | 32 (13.9) | 0.759 |
|  | **Aspirin** | 17 (44.7) | 79 (34.3) | 0.216 |
| Pearson’s chi-square test was used for categorical variables and Independent t-test was used for continuous variables, unless otherwise stated  † Fisher’s exact test  ^‡^ Mann-Whitney test  ^a^ n =64 (23.9%) of data unavailable  ^b^ n=18 (6.7%) of data unavailable  ^¶^ Includes individuals receiving an ACEi or ARB  HbA1c = glycated haemoglobin, SBP = systolic blood pressure, DBP = diastolic blood pressure, BP = blood pressure, TC = total cholesterol, TG = triglycerides, LDL = low density lipoprotein, HDL = high density lipoprotein, BMI = body mass index, eGFR = estimated glomerular filtration rate, UACR = urine albumin creatinine ratio, Hb = haemoglobin, min=minimum value, max = maximum value, ED = emergency department, DPP4i = Dipeptidyl peptidase-4 inhibitor, GLP-1 RA = Glucagon-like peptide 1 receptor agonist, SGLT2i = Sodium glucose co-transporter 2 inhibitor, ACEi = Angiotensin converting enzyme inhibitor, ARB = Angiotensin receptor blocker, CCB = Calcium channel blocker, ESA = Erythropoietin stimulating agent | | | | |
